# Nature and nurture effects on the spatiality of the mental time line

**DOI:** 10.1038/s41598-018-29584-3

**Published:** 2018-08-03

**Authors:** Filomena Anelli, Gregory Peters-Founshtein, Yaen Shreibman, Elior Moreh, Chiara Forlani, Francesca Frassinetti, Shahar Arzy

**Affiliations:** 1 Istituti Clinici Scientifici Maugeri IRCCS, via Maugeri 4, 27100 Pavia, Italy; 2 Neuropsychiatry Lab, Department of Medical Neurobiology, Faculty of Medicine, Hadassah Hebrew University Medical School, Jerusalem, Israel; 3 Department of Neurology, Hadassah Hebrew University Medical Center, 91200 Jerusalem, Israel; 4 Department of Rehabilitation, Hadassah Hebrew University Medical Center, 91200 Jerusalem, Israel; 5 Department of Psychology, University of Bologna, viale Berti Pichat 5, 40127 Bologna, Italy

## Abstract

The nature-nurture debate regarding the origin of mental lines is fundamental for cognitive neuroscience. We examined natural-nurture effects on the mental time line, applying three different challenges to the directionality of time representation. We tested (1) patients with left-neglect and healthy participants, who are (2) left-to-right or right-to-left readers/writers, using (3) a lateralized left-right button press or a vocal mode in response to a mental time task, which asks participants to judge whether events have already happened in the past or are still to happen in the future. Using lateralized responses, a spatial-temporal association of response code (STEARC) effect was found, in concordance with the cultural effects. With vocal responses (no lateralization), past and future events showed similar results in both cultures. In patients with neglect, who have a deficit of spatial attention in processing the left side of space, future events were processed more slowly and less accurately than past events in both cultures. Our results indicate the existence of a “natural” disposition to map past and future events along a horizontal mental time line, which is affected by the different ways in which spatial representation of time is introduced.

## Introduction

Representation of magnitudes plays a major role in our everyday life. Numbers, time and even emotions quantified in our brain, are hypothesized to be represented on “mental lines”^1–3^, spatially relying on a continuous left-to-right orientation axis^2,4,5^. Studies of numerosity in archaic cultures and in children demonstrate a common origin of the mental number line (MNL), that may be modified as based on cultural effects^6,7^. A strong evidence for the association between space and numbers is the so-called Spatial-Numerical Association of Response Codes (SNARC) effect^8^. This effect represents the finding that responses are faster for relatively small numbers while given with the left-hand side and faster for relatively large numbers while given with the right-hand side^9^. For instance, while participants are required to perform an odd–even classification task on numbers from 1 to 9 by pressing a left or right key, responses are faster and more accurate when participants respond with the left key to small numbers and with the right key to large numbers, compared to the opposite instructions^8^. This finding supports the existence of a horizontal spatial representation of numbers along the MNL where, in Western cultures, numbers are progressively positioned from left-to-right (L → R), while a reverse SNARC is reported in right-to-left (R → L) readers/writers^8,10^.

An important contribution to the understanding of the spatial representation of number is provided by neuropsychological studies in brain damaged patients. Such studies demonstrated that patients with a right brain lesion and a contra-lesional visuo-spatial deficit (i.e., left neglect) show a rightward bias in bisecting a number sequence from 0 to 9^11^. This bias was an expression of the deficit in considering the smaller numbers, hypothesized to be represented in the leftmost part of the (horizontal) MNL. However, other studies have found that in patients with right brain damage the ‘rightward’ bias in MNL is not correlated with the attentional bias in visual space^12–15^. Specifically, Aiello and colleagues^16^ found that patients with right hemispheric lesion disregard small numbers both when these are mapped on the left side of the mental number line and on the right side of an imagined clock face. Similar results were found while the right hemisphere was suppressed by electrical stimulation^17^. The hemispheric specialization explanation for the MNL is in line with studies identifying a MNL-like behavior in neonates or animals, in whom cultural-biased cannot account for the number-space effect^18^. These findings have led to the conclusion that the right hemisphere supports the representation of small numerical magnitudes independently from their mapping on the left or the right side of the MNL.

Similarly to the MNL, several studies pointed on the spatial nature of time representation that may be regarded as a mental time line (MTL) (e.g.^1,19–22^; for reviews^5,23,24^). Moreover, a temporal equivalent of the SNARC effect, that is the Spatial TEmporal Association of Response Codes (STEARC) effect, has also been reported. For example, facilitation was found between short temporal durations and left-hand responses, and long temporal durations and right-hand responses^25,26^. A similar effect has also been obtained with temporal concepts: faster responses were found when words with meaning referring to the past were presented on the left and words with meaning referring to the future on the right space^27,28^. Like in the SNARC effect^10,29^, the STEARC effect is prompted by L → R reading and writing direction (left/past and right/future), while a reversed pattern (i.e., left/future and right/past facilitation effect) has been found in R → L readers/writers participants (e.g.^7,30^).

Here, we asked whether the left-right directionality of the MTL is inherent to the cognitive system or, alternatively, whether it emerges only when an explicit horizontal representation is applied. To this aim, we tested participants in a mental time (MT) task, requiring them to determine whether a series of events were located in the past or in the future^1,31–33^. We challenged the habitual STEARC effect using three different modifications of the left-to-right axis: (1) we tested participants who are either L → R or R → L writers and readers; (2) participants responded either with both hands or by vocal response, and (3) we included participants with or without left neglect. These manipulations enabled us to examine the factors that influence the spatial representation of past and future events on the MTL.

## Results

### Experiment 1. Mental time task using manual responses in healthy participants from L → R and R → L cultures

In the mental time (MT) task, brief descriptions of personal (e.g., car license, first child) or non-personal world events (e.g., Obama’s election, Chernobyl disaster) are presented. For each event, participants were required to indicate whether the event had already happened (past event) or is yet to happen (future event)^1,31–33^. In order to examine the directionality of the mental time line (MTL), we first used here a bimodal experimental settings involving 28 participants who are either L → R or R → L readers/writers responding either for past events with the left hand and for future events with the right hand and vice-versa.

### Response times

**Figure 1:**
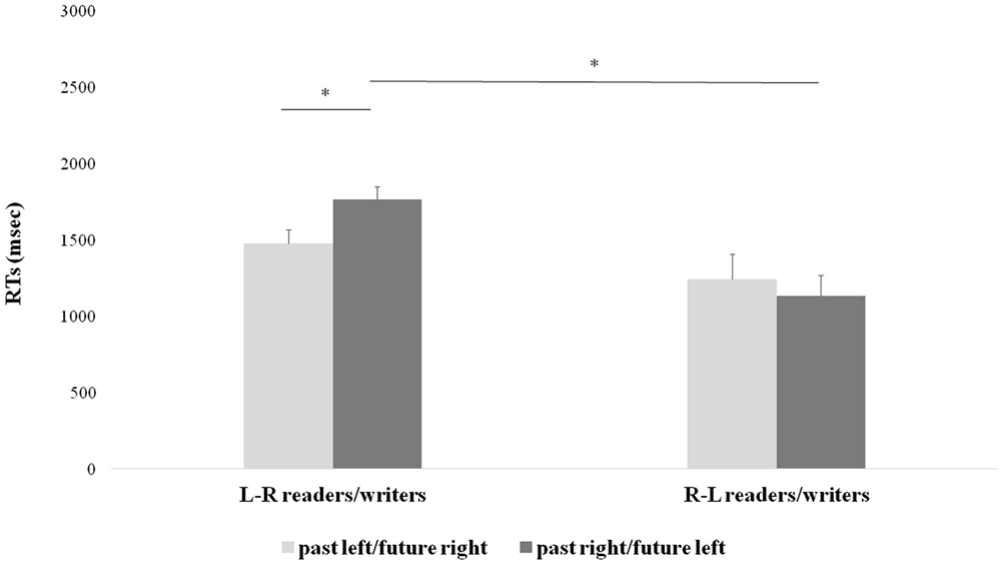
Experiment 1. Significant interaction between *Group* (L → R or R → L readers/writes) and *Laterality* (past/future-left/right) in lateralized responses. In the L → R culture group, responses were faster in the past/left-future/right condition while in the R → L group the other way around was found. Values are indicated in milliseconds (msec); error bars depict standard errors of the mean (SEM); asterisks indicate significant differences (p < 0.05).

Analysis of response times (RTs) showed a main effect of *Group* (L → R vs R → L) [F_(1,26)_ = 7.20, p < 0.05, η^2^_p_ = 0.22], since R → L participants responded faster than L → R participants (mean ± SD, 1188 ± 555 vs 1620 ± 357 msec). Relevantly, the interaction *Group* (L → R vs R → L) × *Laterality* (past/left and future/right vs past/right and future/left) was also significant [F_(1,26)_ = 12.51, p < 0.01, η^2^_p_ = 0.33] (Fig. 1). Duncan post-hoc tests revealed that, in the L → R group, responses were faster in the past/left and future/right condition (i.e., in the condition congruent with L → R culture) with respect to the opposite condition (1476 ± 341 vs 1764 ± 317 msec, p < 0.001; Fig. 1, left). In the R → L group the other way around was found with participants faster for a condition congruent with the R → L culture, though this difference between responses provided in the past/left and future/right and past/right and future/left conditions did not reach significance (1242 ± 613 vs 1134 ± 497 msec, p = 0.18; Fig. 1, right). Moreover, comparing the two groups, the R → L group was faster than the L → R one (1134 ± 497 vs 1764 ± 317 msec, p < 0.01) in responding to the past/right and future/left condition (i.e., in the condition congruent with R → L culture).

### Error rates

The main effect of *Group* (L → R vs R → L) [F_(1,26)_ = 7.55, p < 0.05, η^2^_p_ = 0.23] was significant: R → L participants made more errors than L → R participants (9 vs 4%).

Results of Experiment 1 show that a spatial factor – reading/writing direction – influences judgments related to past and future events, when the MT-task required an explicit spatial coding through left/right lateralized responses. Such space/time association emerged and a facilitation was found when L → R participants responded with the left hand to the past and the right hand to the future, and vice versa for R → L participants, though the latter did not reach significance, hinting on an additional factor in this experimental group (see Discussion for possible interpretations). The present data thus support a culturally-influenced organization of the MTL, where past and future are represented from left-to-right in L → R readers and writers, and vice versa in R → L ones.

Two hypotheses may explain these results. First, there is an intrinsic (“natural”) organization of the MTL (past in the left and future in the right), as it is found for other magnitudes in very young children^18^ and even in animals^34^ (or vice versa). Second, cultural factors influence the directionality of the effects. To allow the spatial representation of time to emerge, in Experiment 1, spatiality was introduced through motor response. In Experiment 2, we tested patients with left-neglect, impaired in the spatial processing of the left visual field^35–40^. Therefore, on the one hand we removed the spatial dimension from the MT-task (using vocal instead of motor response), on the other hand we introduced space in the group of neglect patients due to their spatial deficit. Following a right brain lesion, neglect patients show a disorder characterized by an unbalanced distribution of attention in space, with a deficit in orienting attention to the contralesional space and an attentional bias for the ipsilesional space. Based on this view, two opposite results can be predicted. According with the deficit in orienting attention towards stimuli in contralesional space^41^, a significant impairment in representing events associated with the left portion of space is expected (i.e., slower response times and higher error rate to past events, assuming a L → R MTL). Instead, if neglect reflects an ipsilesional attentional bias, consisting of enhanced attention to the right^42^, patients should spend more time in exploring the right space where future events are located (i.e., slower response times and higher error rate to future events, assuming a L → R MTL). If natural effects are prominent compared to cultural ones, the above reported scenarios should affect both L → R and R → L readers/writers neglect patients in the same way while vocal responses are given. If the reading/writing directionality plays instead a key role, left neglect will affect the performance of L → R and R → L readers/writers differently.

### Experiment 2. Mental time task using vocal responses in healthy participants and patients with left neglect from L → R and R → L cultures

To distinguish between these two hypotheses, in Experiment 2 participants were required to give vocal (i.e., non-lateralized) responses to past/future events. Moreover, we verified whether, without a lateralized motor response, the spatial encoding of past and future events emerged when an attentional spatial deficit is present, as happens following a brain lesion in neglect patients, and how this interacts with cultural factors. To this aim, in Experiment 2, we examined 7 left-neglect patients (NP) and 7 healthy controls (HC) who are solely L → R readers and writers, and 7 NP and 7 HC who are solely R → L readers/writers. In addition to the audio version of the MT-task, we performed lesion analysis to ensure homogeneity between the two patients groups.

### Lesion Analysis

**Figure 2:**
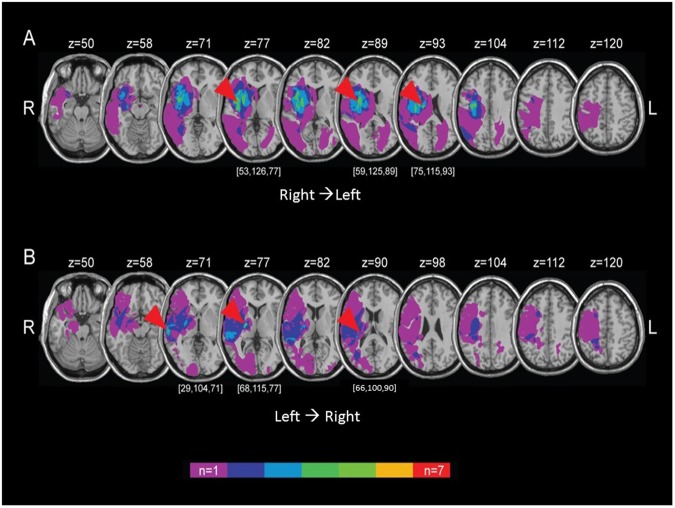
Lesion analysis. The spatial distribution of ischemic lesions (transformed into standard MNI stereotaxic space) in all R → L (**A**) and L → R (**B**) patients is demonstrated by overlaying the lesion masks onto a T1-weighted atlas. Voxels are color-coded in accordance with the number of patients with a lesion affecting a specific voxel. Arrowheads are directed at foci of lesion map convergences (x, y, z coordinates denoted in brackets).

The lesion overlay of R → L patients revealed three primary regions of overlap: in the *right putamen and claustrum* (x = 53, y = 126, z = 77) appearing in four out of the seven patients, at the *right insula* (x = 59, y = 125, z = 89) appearing in five patients, and in the *right caudate* (x = 75, y = 115, z = 93) appearing in four patients, such that every R → L patient exhibited a lesion in at least one of these three foci (Fig. 2A). The lesion overlay of the L → R patients revealed two primary foci of convergence: five out of the seven patients exhibited a lesion in the *right posterior limb of the internal capsule* (x = 68, y = 115, z = 77) and four patients exhibited a lesion in the *right middle temporal gyrus* (x = 29, y = 104, z = 71), such that every L → R patient exhibited a lesion in at least one of these two foci (Fig. 2B). Taken together, underlying lesions in most patients from both R → L and L → R cultures were located in the right basal ganglia, as well as the right temporal/insular cortex. The spatial distribution of lesions is within the normal range for neglect patients, since the most frequent sites of damage in the right hemisphere involve temporal cortex and subcortical nuclei, along with inferior parietal lobule and ventral frontal region^43,44^.

### Behavioural Analysis

#### Response times

**Figure 3:**
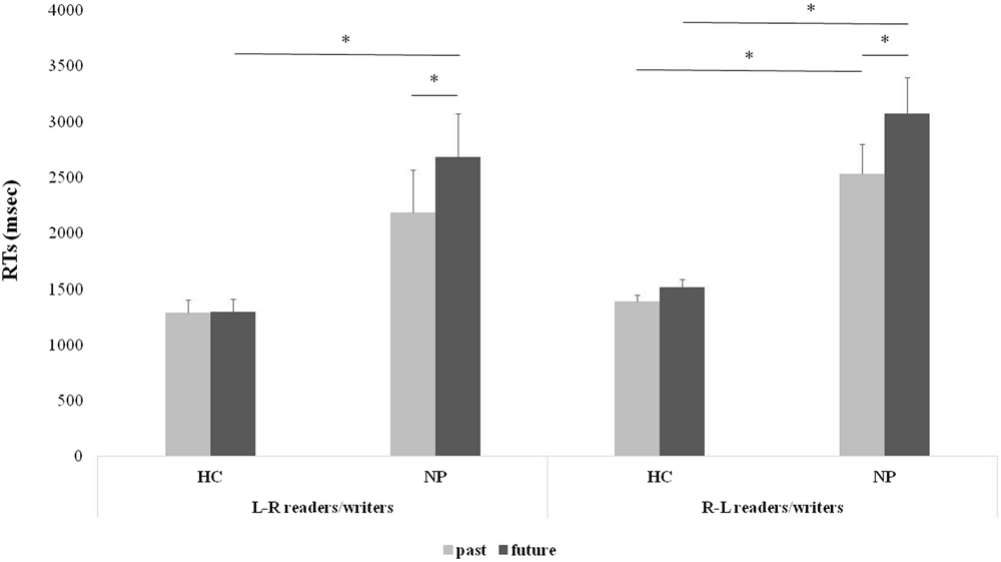
Experiment 2. Significant interaction between *Group* (L → R or R → L readers/writes) and *Time* (past/future) in non-lateralized responses. Similar results were found in both the L → R and R →  L culture groups: responses were faster in healthy controls (HCs) than in neglect patients (NPs). Note the similarity of response times between the two groups, as in NPs in both cultures future responses were slower than past ones. Values are indicated in milliseconds (msec); error bars depict standard errors of the mean (SEM); asterisks indicate significant differences (p < 0.05).

The main effects of *Group* (L → R HC, R → L HC, L → R NP, and R → L NP) [F_(3,24)_ = 8.79, p < 0.001, η^2^_p_ = 0.49] and *Time* (past vs future) [F_(1,24)_ = 44.64, p < 0.001, η^2^_p_ = 0.65] were significant, as well as their interaction [F_(3,24)_ = 9.27, p < 0.001, η^2^_p_ = 0.54] (Fig. 3). Both L → R and R → L control groups did not show a significant difference between responses to past and future events (L → R HC 1286 ± 306 vs 1292 ± 301 msec, p = 0.94; R → L HC 1389 ± 157 vs 1514 ± 173 msec, p = 0.16). Conversely, both neglect patients groups were significantly slower in responding to future than to past events (L → R NP 2680 ± 1022 vs 2181 ± 1013 msec, p < 0.001; R → L NP 3066 ± 868 vs 2529 ± 708 msec, p < 0.001). Overall, R → L NPs responded slower than respective HC in both past and future events (p < 0.05 for both comparisons), and L → R NPs were slower than respective HC in particular when considering future events (p < 0.05).

#### Error rates

Overall, error rates were slightly higher for future than for past events (6% vs 4%), though main effect of *Time* (past and future) did not reach significance [F_(1,24)_ = 3.30, p < 0.08] and the interaction *Group* × *Time* was not significant (p = 0.16). Means showed that both patients groups made more errors for future than past responses (L → R NPs 10% vs 5%; R → L NPs 10% vs 6%) whereas this was not the case for healthy controls (L → R HCs 1% vs 2%; R → L HCs 1% vs 1%).

#### Crawford’s test

The previous analyses revealed that both neglect patients groups were significantly slower in responding to future than to past events. We conducted an additional analysis to verify whether this effect was limited to a few patients or, alternatively, whether it emerged in most of our patients. To this aim, we used the Crawford’s test^45^, a modified t-test, to compare the RTs of each NP with respective HC. The mean values suggest that this effect was present in all patients and the Crawford’s test was significant in 5 out of 7 L → R NP and in 6 out of 7 R → L NP (p < 0.05).

Overall, our data highlight the role played by spatial factors in the emergence of spatio-temporal association. The STEARC effect emerges only when a spatial factor is involved, such as when responses are given in a lateralized manner (Experiment 1) or when an attentional spatial bias is present, as happens in left neglect patients (Experiment 2). These findings are discussed below in the framework of attentional influence on the MTL.

## Discussion

In the present study, we tested factors that influence the MTL and its directionality, requiring participants to determine whether a series of events were located in the past or the future. Three different factors were checked: response modality (motor vs verbal), cultural influence (L → R vs R → L reading/writing direction), and spatial attention (participants with vs without left neglect). In healthy participants, we found a spatial-temporal association of response codes (STEARC) effect in concordance with reading/writing direction when responses were given in a lateralized manner (motor MT-task). In left-neglect, the left-right axis is interrupted following a spatial attention deficit, and a significant difficulty for future processing has been found, in terms of both slower response times and higher error rate, in line with previous studies^46^. Notably, similar results in processing past and future events in healthy participants elicited by Experiment 2 (vocal MT-task) speak against the possibility that uncontrolled factors could make future events intrinsically more difficult than past events.

In Experiment 1, healthy participants showed facilitation in responding to past stimuli with the left key and to future ones with the right key in L → R culture (STEARC effect). The opposite pattern (facilitation for future stimuli while responded with the left key and to past ones with the right key) was found in R → L culture. These results are in line with the interpretation of the STEARC effect as related to a conflict emerging at the stage of response selection (e.g.^26^), in analogy with similar effects found in other domains, like sound pitches (SMARC effect^47^) or numbers (e.g., SNARC effect^8^). For example, regarding the SNARC effect, and in accordance with findings by Gevers and colleagues^48^, this conflict emerges when two alternative response codes are possible: one automatically triggered through pre-existing associations linking a stimulus to its response, and the other generated on the basis of task instructions (dual-route account; e.g.^49^). If both routes converge on the same response, a response can be activated fast; conversely, if the two routes elicit different responses, the automatic response should be inhibited before the correct response can be given.

According to these theories, the effect found here can be explained by a conflict between responses elicited by a culture-dependent time representation and task-related responses. Indeed, facilitation was registered when L → R participants responded with the left hand to the past and the right hand to the future, and vice versa for R → L participants. However, this latter effect did not reach significance probably because R → L readers/writers participants in Experiment 1 were also exposed to the opposed direction of language and mathematical schemes (see also^30,50,51^). Note that “hybrid” reading/writing habits for text and numbers is known to decrease the SNARC effect^10,52^ as may be the case also for time^53^. Alternatively, weaker effect in R → L participants may suggest that there is an intrinsic (“natural”) organization of the MTL (past in the left and future in the right), that can be influenced but not overwritten by cultural habits. This directionality from left to right of past and future events is in line with previous studies (for review see^5^).

In Experiment 2, when a lateralized response was not required and a verbal response was given, past and future events were processed similarly by healthy participants. Space-time association was found by several studies^25–27,33,54–57^. More in detail, such a space-time association is present when responses are given in a lateralized manner by a manual response code (Experiment 1 reported here), when there is a disruption of lateralization as occurs following left neglect (Experiment 2 reported here, see also^46^), and when a lateralization is induced by shifting visuo-spatial attention through prismatic adaptation^33^. In this latter study, we showed that prism-induced manipulations of spatial attention influenced processing of past and future concepts. In particular, we obtained a facilitation in responding to past/future events according to the direction of the attentional shift, by using a verbal/non-lateralized response (as in Experiment 2 reported here): leftward and rightward shift of spatial attention facilitated indication of past and future events, respectively. Thus, our data highlight how spatial attention prioritizes processing of particular locations in time, which failed to emerge before prismatic modulation, as shown here in Experiment 2.

In order to eliminate the hand-related effect (i.e., removing space from the MT-task), we used here audio presentation of stimuli and vocal response modes, which have proven efficient in our previous studies^33,46^. Notably, in Experiment 2 the lack of effects in healthy participants is due to the experimental manipulation that, through acoustical binaural presentation and vocal responses, made space not a dimension of the task. Similarly, Magnani and colleagues^58^ found that a spatial representation of auditory time (i.e., in a task where participants classified the duration of auditory stimuli as “short” or “long”) emerges selectively when space becomes a relevant dimension, enforcing a spatial-encoding of auditory stimuli. More specifically, in their experiment an underestimation of the duration of auditory stimuli presented on the left, relative to that presented on the right, was found only when participants were first required to localize stimuli. Taken together, the current as well as previous results point on the emergence of a space-time association (i.e., a horizontal spatial representation of time) under certain conditions requiring lateralization.

Regarding the alteration of the MTL following a spatial attention impairment in patients with left-neglect, we found longer response times and higher error rates for future events in patients from both L → R and R → L cultures. Several explanations may account for these findings. The first explanation assumes a MTL oriented from right-to-left. Both patients’ group with left neglect (L → R and R → L) faced difficulty in processing future events, suggesting the future to lie in the disturbed side, namely the left. However, this hypothesis is not in-line with previous studies suggesting a L → R MTL^5^,^39^,^40^, though most of these studies adopted an allocentric point of view while the current study adopts an egocentric one. The difference in significance between L → R and R → L participants in Experiment 1 also suggests a L → R MTL.

An alternative interpretation of our results can be provided by the hemispheric-specialization theory proposed in the domain of number representation. According to this theory, the right hemisphere represents small numerical magnitudes and the left hemisphere represents large magnitudes, independently from their mapping on the left or the right side of the mental number line^16,34^. Following an analogous mental time and mental number lines, future impairment in our patients implies that the right hemisphere accounts for future processing and the left hemisphere for the past.

A third explanation is related to the potential character of the future vs real events in the past. Future events, as used here, can be regarded as potential events which might never happen, while past events can be labelled as factual events which have surely occurred in the past. Thus, our MT-task may require participants to judge whether something is factual or potential. The difficulty of neglect patients in processing future events can be in line with a recent study revealing that participants were faster in responding to potential events with the left hand and to factual events with the right hand, than with the opposite mapping^59^. The dimension of factuality-potentiality may therefore be also mapped onto space, similarly to time and number, with a left-potential and right-factual mapping. This may lead to impairment in potential-events processing in our patients.

A final explanation refers to the hypothesis that neglect reflects a rightward attentional bias^42^: neglect patients would spend more time in exploring the right space than left space^33^. Assuming a L → R MTL, longer and pathological exploration of the right-space would lead to slower response times to future events^46^. Though longer exploration may reduce errors, higher error rate found here may be related to false recognitions.

In conclusion, our results suggest that spatial components may interfere with the processing of time. This interference may be further influenced by other factors such as reading/writing direction. Additional research may explore such hierarchical nature-nurture influences on the MTL and other mental lines, and provide theoretical and experimental frameworks for their potential interpretations.

## Methods

### Experiment 1

#### Participants

28 right-handed healthy participants (mean age ± SD: 62 ± 6 years old) took part in Experiment 1, half of them L → R readers/writers (Italian speakers) and half R → L readers/writers (Hebrew speakers living in Israel who were also exposed to L → R language and mathematical schemes). Participants did not show any cognitive impairment as measured by the Mini Mental State Examination (MMSE score ≥29)^60^. All participants were right-handed, had normal or corrected-to-normal vision, and no history of psychiatric diseases. Participants involved in all reported experiments were naive to the purpose of the study and provided written informed consent. All the reported experiments were carried out in accordance with the 2008 Helsinki Declaration and approved by the Ethical Committees of Hadassah Medical Center and the University of Bologna.

#### Stimuli and procedure

**Figure 4:**
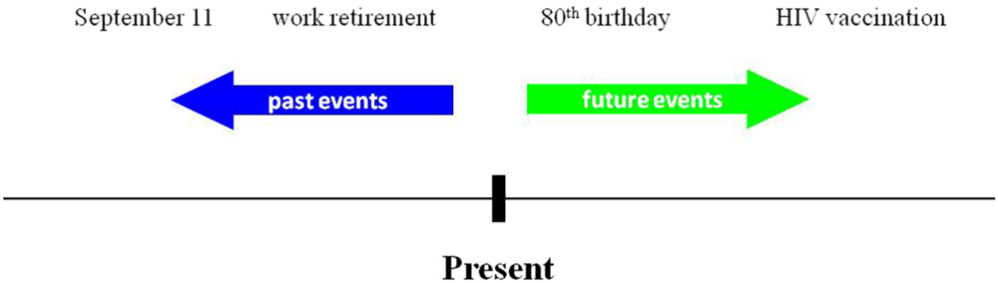
The mental time task. Participants were presented with personal or non-personal life events. They had to determine whether an event has already happened in the past or is expected to happen in the future.

During the MT-task, participants sat in front of a 15-inch color monitor, at a distance of about 60 cm. Brief descriptions of personal (e.g., car license, first child) or non-personal world events (e.g., Obama’s election, Chernobyl disaster) were presented on the computer screen (similar to^1,31–33^), including 24 stimuli (half personal and half non-personal), equally distributed between past and future events (Fig. 4; for a complete list of stimuli, see Table 1). For each event, participants were required to indicate whether the event had already happened (past event) or was yet to happen (future event).

**Table 1:** List of events presented to participants (Experiments 1 and 2).

Personal events (relative past)	Using glasses
	40^th^ birthday
	First child birth
	First car purchase
	Retirement from work
	First hospitalization
Personal events (relative future)	80^th^ birthday
	First great grandchild
	Grandchild marriage
	Diamond wedding
	Admission to nursing home
	Child retirement
Non-personal events (relative past)	Obama’s election
	First use of Euro
	Chernobyl disaster
	Man on the moon
	September 11
Non-personal events (relative future)	Peace in Middle East
	Defeat illnesses
	Woman president in USA
	Defeat mafia
	HIV vaccination
	Defeat world hunger

Each stimulus appeared in the center of the computer screen and remained visible until a response was given, with an inter-stimulus interval of 1000 msec. The experiment had two blocks, differing in the mapping of the left and right keys to “past” or “future” judgments (i.e., mapping 1 past events/left key and future events/right key; mapping 2 past events/right key and future events/left key; motor MT-task). The order of blocks was counterbalanced over participants (within-participants design). E-Prime 2.0 software was used for stimulus presentation and response collection.

### Statistical analysis

Data on response times (RTs) and error rates (ERs) were entered in separate repeated-measures analysis of variance (ANOVA) with *Time* (past vs future) and *Laterality* (past/left and future/right vs past/right and future/left) as within-subject factors, and *Group* (R → L vs L → R) as between-subject factor. Duncan post-hoc tests were performed on significant interactions and the magnitude of effect size was expressed by η^2^_p_.

### Data availability

The datasets generated and/or analyzed during the current study are available from the corresponding author on reasonable request.

### Experiment 2

#### Participants

7 left-neglect patients (NP) and 7 healthy controls (HC) who are solely L → R readers and writers (Italian speakers), and 7 NP and 7 HC who are solely R → L readers/writers (Hebrew or Arabic speakers) participated in Experiment 2 (mean age ± SD: 66 ± 7.84 years old, for neuropsychological details see Table 2). All patients were recruited for the experiment within 6 months from the onset of illness. The day before the experimental session, all patients were submitted to Mini Mental State Examination (MMSE) and a battery of tests assessing the presence of neglect (Behavioral Inattention Test Battery – conventional part, star cancellation, and line bisection) assuring that all patients had an active left-neglect at the time of testing (Table 2). Healthy participants and patients did not show significant cognitive impairment. L → R patients with a right brain lesion and without neglect have also been tested and showed the same pattern of results of controls [46; see also Supplementary Information], excluding that the difference between neglect patients and controls can be explained by a brain lesion *per se* or by differences in past and future events related to uncontrolled effects.

**Table 2:** Summary of clinical data for patients with left neglect (NP).

Group	Sex	Age (years)	Education (years)	BIT-C (cut-off 129)	Star cancellation test (cut-off 51)	Line bisection (cut-off 1,07 +/− 4,66)
L → R NP 1	F	82	10	**8**	**3**	**7,92**
L → R NP 2	M	56	8	**126**	**49**	NA
L → R NP 3	M	76	17	**127**	**44**	−1,74
L → R NP 4	F	43	16	**59**	**31**	NA
L → R NP 5	F	73	5	**56**	**16**	NA
L → R NP 6	M	75	18	**108**	**38**	0,15
L → R NP 7	M	64	11	**71**	**18**	**7,25**
R → L NP 1	M	60	15	**86**	**12**	**5,6**
R → L NP 2	M	66	12	**118**	**50**	**1**
R → L NP 3	M	67	18	NA	**9**	**3**
R → L NP 4	F	64	10	**59**	**41**	**1,5**
R → L NP 5	M	72	18	NA	**50**	**1**
R → L NP 6	M	68	12	NA	**46**	**2**
R → L NP 7	M	63	16	**115**	**15**	**2**

Values below the cut-off are reported in bold. NA = data not available; BIT-C = BIT conventional.

#### Stimuli and procedure

The stimuli and procedure were the same as outlined in Experiment 1, except that here participants were presented with audio stimuli and provided a vocal response (“past” vs. “future”; verbal MT-task). This response modality removes spatialization of stimulus’ representation (for the same procedure, see^33^). Patients with neglect were not tested using motor responses due to left hand weakness.

#### Lesion analysis

Lesion tracing was completed using MRIcron software (http://people.cas.sc.edu/rorden/mricron/index.html). Lesions were visually identified as having altered signal intensity in relation to equivalent contralateral tissue. Lesions were traced by a trained image analyst and confirmed by an experienced neurologist. Brain images and lesion tracings were normalized to standard stereotaxic space (MNI space), using an extension of the Statistical Parametric Mapping (SPM) software, named “Clinical Toolbox”, that transforms a lesion image to a dilated lesion mask, applies it to its corresponding brain image to ensure normalization is driven by intact brain tissue^61,62^, and outputs a normalized binary lesion map as well as the normalized CT/MRI image. Lesion maps were superimposed, and the extant of overlap between patients was estimated and projected onto a T1 template MNI image.

#### Behavioural analysis

Data on RTs and ERs were entered in separate repeated-measures ANOVAs with *Time* (past vs future) as within-subject factor, and *Group* (L → R HC, R → L HC, L → R NP, and R → L NP) as between-subject factor. Duncan post-hoc tests were performed on significant interactions and the magnitude of effect size was expressed by η^2^_p_.

## Electronic supplementary material

Supplementary Information
