# Supplementary material for: Nature and nurture effects on the spatiality of the mental time line
Source: Sci Rep. 2018 Aug 3;8:11710. doi: 10.1038/s41598-018-29584-3 (PMC6076263; doi:10.1038/s41598-018-29584-3)
Supplement: Supplementary file 1 — Supplementary Information [file 41598_2018_29584_MOESM1_ESM.pdf]

Nature and nurture effects on the spatiality of the mental time line

Filomena Anelli[1*°], Gregory Peters-Founshtein [2,3°], Yaen Shreibman[2], Elior Moreh[4], Chiara Forlani[1], Francesca Frassinetti[1,5] and Shahar Arzy[2,3]

[1] *Istituti Clinici Scientifici Maugeri IRCCS, via Maugeri 4, 27100 Pavia, Italy*

[2] *Neuropsychiatry Lab, Department of Medical Neurobiology, Faculty of Medicine, Hadassah Hebrew University Medical School, Jerusalem, Israel*

[3] *Department of Neurology, Hadassah Hebrew University Medical Center, 91200 Jerusalem, Israel*

[4] *Department of Rehabilitation, Hadassah Hebrew University Medical Center, 91200 Jerusalem, Israel*

[5] *Department of Psychology, University of Bologna, viale Berti Pichat 5, 40127 Bologna, Italy*

° Equal contribution

Supplementary Information

Mixed linear models

Experiment 1

We constructed two mixed linear models in order to test the relationship between space/time associations and response time. Response time (RT) was considered the outcome. Space/time association (past-left/future-right and past-right/future-left) and time of stimuli (future or past) were considered fixed factors. Random effects consisted of individual subject identities as intercepts and by-subjects random slopes for the both fixed effects. Random effects were introduced to account for the individual differences in subjects baseline response times (intercept) as well as for the potential individual differences in the effects of the fixed factors on response time (slopes). This procedure was repeated separately for R→L and L→R subjects, resulting in two models specified by the formula:

$$\text{Response time} \sim 1 + \text{space/time associations} + \text{time of stimuli} + (1 + \text{space/time associations} + \text{time of stimuli} \mid \text{subject identity})$$

P-values were obtained by likelihood ratio tests of the full model with the effect in question against the model without the effect in question.

For R→L subjects, the model demonstrates that Space/time association and time of stimuli has no significant effect on response time:

Fixed effects coefficients (95% confidence interval):				
Name	Estimate	Standard Error	$\chi^2(1)$	p-value
Intercept	2.48	0.3	67.501	<0.001
Space/time association	-0.2	0.18	1.1875	0.27
time of stimuli	0.08	0.15	0.2647	0.60
Random effects parameters (95% confidence interval):				
Name	Estimate	Lower	Upper	
Subject identity intercept	1.03	0.69	1.54	

By-subject space/time association slope	0.42	0.16	1.12	
By-subject time of stimuli slope	0.10	0.007	1.64	
Residual	1.71	1.61	1.82	

For L→R subjects, the model demonstrates that both Space/time association and time of stimuli has significant effect on response time:

Fixed effects coefficients (95% confidence interval):				
Name	Estimate	Standard Error	$\chi^2(1)$	p-value
Intercept	1.53	0.08	306.68	<0.001
Space/time association	0.29	0.05	26.229	<0.001
time of stimuli	-0.12	0.05	6.6728	0.01
Random effects parameters (95% confidence interval):				
Name	Estimate	Lower	Upper	
Subject identity intercept	0.29	0.19	0.43	
By-subject space/time association slope	0.12	0.042	0.35	
By-subject time of stimuli slope	0.04	0.004	0.52	
Residual	0.57	0.54	0.61	

Experiment 2

We constructed two mixed linear models in order to test the relationship between clinical status and response time. Response time (RT) was considered the outcome. Clinical status (neglect and non-neglect) and time of stimuli (future or past) were considered fixed factors. Random effects consisted of individual subject identities as intercepts and by-subjects random slopes for the both fixed effects. Random effects were introduced to account for the individual differences in subjects baseline response times (intercept) as well as for the potential individual differences in the effects

of the fixed factors on response time (slopes). This procedure was repeated separately for R→L and L→R subjects, resulting in two models specified by the formula:

$$\text{Response time} \sim 1 + \text{clinical status} + \text{time of stimuli} + (1 + \text{clinical status} + \text{time of stimuli} \mid \text{subject identity})$$

P-values were obtained by likelihood ratio tests of the full model with the effect in question against the model without the effect in question.

For R→L subjects, the model demonstrates that clinical status but not time of stimuli had significant effect on response time:

Fixed effects coefficients (95% confidence interval):				
Name	Estimate	Standard Error	$\chi^2(1)$	p-value
Intercept	2.73	0.17	246.43	<0.001
Clinical status	2.47	0.41	35.49	<0.001
Time of stimuli	0.22	0.22	0.93	0.33
Random effects parameters (95% confidence interval):				
Name	Estimate	Lower	Upper	
Subject identity intercept	0.67	0.40	1.12	
By-subject clinical status slope	0.98979	0.51146	1.91	
By-subject time of stimuli slope	0.21	0.02	2.4	
Residual	1.76	1.63	1.92	

For L→R subjects, the model demonstrates that clinical status and time of stimuli had a significant effect on response time:

Fixed effects coefficients (95% confidence interval):				
Name	Estimate	Standard Error	$\chi^2(1)$	p-value

Intercept	1.36	0.11	148.73	<0.001
Clinical status	1.64	0.46	12.75	<0.001
time of stimuli	-0.21	0.10	4	0.04
Random effects parameters (95% CIs)				
Name	Estimate	Lower	Upper	
Subject identity intercept	0.86	0.58	1.27	
By-subject clinical status slope	1	0.46	2.19	
By-subject time of stimuli slope	0.11	0.03	0.18	
Residual	0.92	0.84	0.99	

All analysis were performed using Matlab-based scripts (MATLAB version 17a, MathWorks)

Additional Behavioural Analyses – Experiment 2

In order to verify whether the difference between neglect patients and controls can be explained by a brain lesion *per se*, a group of 7 patients without neglect (N-P) who are solely L→R readers and writers (mean age ± SD: 64 ± 10.4 years old) with a right brain lesion and without neglect have also been tested.

Data on RTs and ERs were entered in separate repeated-measures ANOVA with *Time* (past vs future) as within-subject factor, and *Group* (L→R HC, R→L HC, L→R NP, R→L NP, and L→R N-P) as between-subject factor. Duncan post-hoc tests were performed on significant interactions and the magnitude of effect size was expressed by η^2_p.

Response times

The main effects of *Group* (L→R HC, R→L HC, L→R NP, R→L NP, and L→R N-P) [$F_{(4,30)}$ = 8.22, $p < .001$, η^2_p =.52] and *Time* (past vs future) [$F_{(1,30)}$ = 30.16, $p < .001$, η^2_p = .50] were significant, as well as their interaction [$F_{(4,30)}$ = 583, $p < .001$, η^2_p =.44] (Fig. 1S). Both L→R and R→L control groups, as well as right brain damaged patients without neglect, did not show a significant difference between responses to past and future events (L→R HC 1286 ± 306 vs 1292

± 301 msec, p = .94; R→L HC 1389 ± 157 vs 1514 ± 173 msec, p = .16; L→R N-P 1735 ± 209 vs 1825 ± 341 msec, p = .39). Conversely, both neglect patients groups were significantly slower in responding to future than to past events (L→R NP 2680 ± 1022 vs 2181 ± 1013 msec, p < .001; R→L NP 3066 ± 868 vs 2529 ± 708 msec, p < .001). Overall, R→L neglect patients responded slower than respective healthy controls in both past and future events (all ps < .05 for both comparisons), and L→R neglect patients were slower than respective healthy controls in particular when considering future events (p < .05). Furthermore, both L→R and R→L neglect groups were slower in processing future events (p = .08 and p < .05, respectively), but not past events (both p > .16), also with respect to patients without neglect. Finally, performance of patients without neglect did not differ from both L→R and R→L healthy controls' performance (all p > .30).

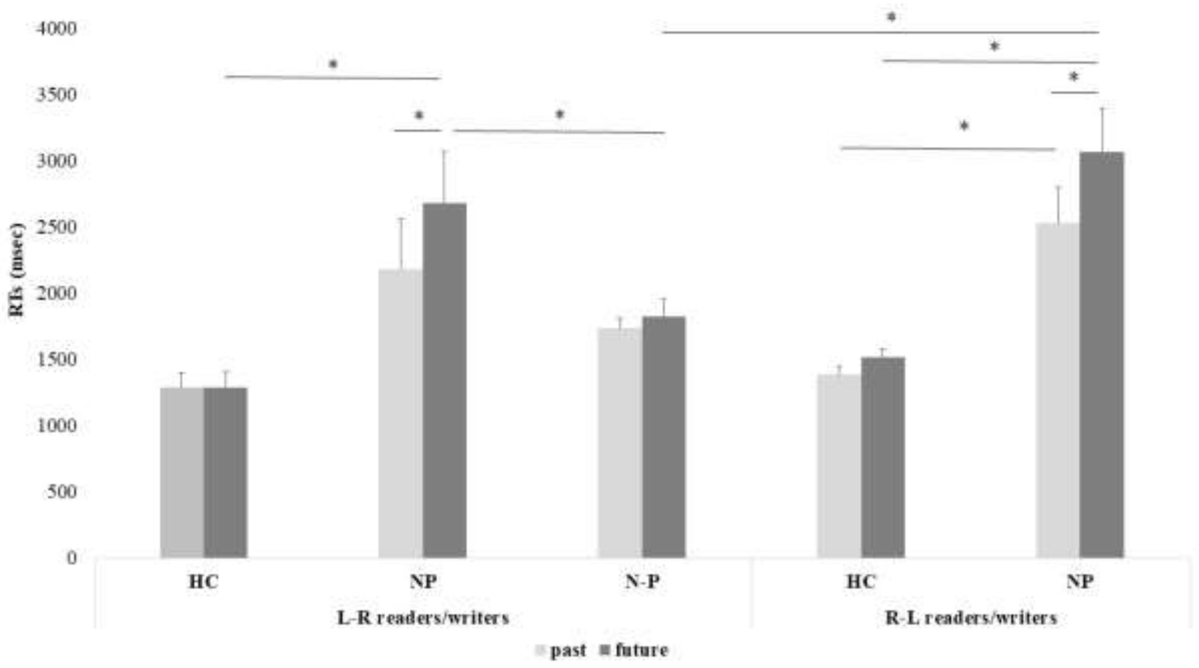

Fig. 1S. Supplementary results. Significant interaction between *Group* and *Time* (Experiment 2). In the neglect patients (NP) in both cultures, future responses were slower than past ones and differed from future responses reported by healthy controls (HC) and patients without neglect (N-P) groups. Values are indicated in milliseconds (msec); error bars depicted standard errors of the mean (SEM); asterisks indicate significant differences (p < 0.05).

Error rates

Overall, error rates were higher for future than for past events (6% vs 4%), as indicated by the main effect of *Time* (past vs future) [$F_{(1,30)} = 3.95$, $p = .05$], while the interaction *Group* x *Time* was not significant ($p = .30$). Means showed that both neglect groups made more errors for future than past responses (L→R NP 10% vs 5%; R→L NP 10% vs 6%) whereas this was not the case for healthy controls (L→R HC 1% vs 2%; R→L HC 1% vs 1%), nor for patients without neglect (L→R N-P 6% vs 4%).
